# Medical and Philosophical News

**Published:** 1781-11

**Authors:** 


### SECTION III.

### Medical and Philosophical News.

THE Royal Academy of Sciences at Rouen have propoſed the following queſtion for a prize of three hundred livres. "In what "degree, and in what manner can we depend "on magnetiſm, or electricity (either poſitive "or negative) in the treatment of diſeaſes?" The diſſertations on this ſubject are to be ſent to M. Dambourney at Rouen before July 1, 1782.

---

Extract of a letter from an ingenious practitioner at Sandwich in Kent, to a Member of the Society, dated October 10, 1781.

"My druggiſt, Hopkins of Paternoſter Row, "has introduced among us a new kind of bark,

"which

"which is of a brick-duſt colour when powder-"ed. It has done wonders in the intermittents, "which have been uncommonly troubleſome "this autumn, and would not give way to the "beſt common bark, unleſs given in enormous "doſes. You may be aſſured that it is infinitely "ſuperior to the grey bark, and ſhould be ſought "after by practitioners."— On inquiry we find, that the bark here ſpoken of was part of the cargo of a Spaniſh prize. Mr. Hopkins purchaſed thirty cheſts of it, twenty-ſeven of which have been already diſtributed among his cuſtomers in Eſſex and Kent, all of whom agree in extolling its ſuperior efficacy in the cure of intermittents.

---

In our Journal for Auguſt we promiſed to give ſome account of the eulogies of the late Baron Haller and M. de Jussieu, publiſhed in the *Memoirs of the Academy of Sciences at Paris* for 1777. To copy the whole of thoſe papers would be to exceed the limits of our plan. We ſhall therefore ſelect from them the following anecdotes of the eminent perſons above-mentioned.

Of the celebrated Haller, his biographer relates, that his genius began to diſplay itſelf at a very a very early period. When he was only nine years old he compofed a Chaldee grammar, a Greek and Hebrew dictionary, and an hiftorical dictionary which contained upwards of two thoufand articles, abridged from Moreri and Bayle.

This learned man paffed almoft the whole of his life in ftudy. His wife, his children, all affifted him in his labours, by extracting paffages from books, or making drawings of plants or animals. As a proof of his activity we are told, that having had the misfortune to break his right arm, the furgeon who vifited him was furprized the next day to find him writing with his left hand.

His life was embittered by domeftic misfortunes. His firft wife died a month after her arrival at Gottingen, of a mifcarriage occafioned by a fall. He vented his grief in two beautiful odes, which excited the fenfibility of all the admirers of German poetry. Two years after this he married again, and his wife died at the end of a few months. He lamented this fecond wife in the fame manner as he had done the firft; and the world, obferves the hiftorian, perceiving how eafily he found a confolation in poetry, began not to pity him. His third wife, who who ſurvives him, was the daughter of Dr. Teichmeyer, profeſſor of anatomy at Jena.

From 1753, the year in which he quitted Gottingen, to the time of his death, he reſided at Berne, his native city, where he took an active part in public buſineſs as a magiſtrate. He improved the ſalt manufactory, and prevailed on government to increaſe the ſalaries of the clergy in the Pays de Vaud. The inhabitants of Berne are likewiſe indebted to him for the inſtitution of an orphan houſe. His experiments on the formation of the chick, his great work on phyſiology, and his Bibliotheca, were the chief of his philoſophical labours during the ſame period.

For ſome years before his death he had a painful affection of the bladder, the ſeverity of which he mitigated by opium. In the midſt of his ſufferings from this diſeaſe, he employed himſelf in completing his Bibliotheca, and in preparing a new edition of his phyſiology for the preſs. When he found his health declining, he requeſted Dr. Roſſelet, his phyſician, to conceal nothing from him. His friend had the courage to tell him his real ſentiments of his caſe. Haller, when he perceived his laſt moments approaching, felt his pulſe from time to time; and and at laſt turning to Roſſelet, "My dear friend —ſaid he—the artery has done beating," and the next moment expired. He was born on the 16th of October 1708, and died Dec. 12, 1777, leaving four ſons and four daughters.

Bernard de Jussieu, M. D. Member of the Academy of Sciences at Paris, &c. was the ſon of an apothecary at Lyons, where he was born on the 17th of Auguſt 1699. He came to Paris in 1714 to finiſh his ſtudies under his elder brother, Anthony, who was at that time a celebrated profeſſor of botany. With this brother, in 1716, he undertook a botanic journey to the Pyrenèes, Spain and Portugal. On his return he viſited a part of the Alps, and then went to ſettle as a phyſician at Lyons; but it was not long before he returned to the capital to ſucceed Vaillant, who reſigned the place of demonſtrator of botany in his favour. The king's garden and muſeum of natural hiſtory were at that time in a neglected ſtate, but M. de Juſſieu ſoon reſtored them to good order.

He was a complete maſter of every branch of natural hiſtory, and at the meetings of the Academy of Sciences, at which he was a conſtant attendant for the ſpace of fifty years, his opinion was always deciſive on matters of that ſort.

Lewis

Lewis XV. employed him in forming a botanic garden at Trianon, and was fond of his converſation, but that prince never made him any recompence for his trouble, nor even reimburſed him the expence he was at in his frequent journies on that buſineſs. Happily his elder brother, by leaving him a good fortune, had rendered him independent.

M. de Juſſieu viſited England twice, and brought from thence the firſt cedar of Lebanon that was planted in France. His life was uniformly ſpent in acquiring and diſſeminating natural knowledge. The diffidence he entertained of his abilities prevented him from publiſhing many works, but no man was ever more ready to communicate his knowledge. Pupils reſorted to him from every country, and all the great botaniſts of Europe conſidered him as their maſter.

On the 20th of September 1777, he was ſtruck with apoplexy, after which he languiſhed till his death, which happened on the 6th of November following. Dying unmarried, he bequeathed the bulk of his fortune to one of his nephews, who is a phyſician of conſiderable eminence at Paris.

Beſides Anthony de Juſſieu already mentioned, another of his brothers, Dr. Joſeph de Juſſieu, who who died lately at Paris, acquired ſome celebrity as a phyſician and naturaliſt, and was one of the French academicians ſent by Lewis XV. to Quito to Spaniſh America, in order to aſcertain the figure of the earth.

